# Supplementary material for: Exploring Host-Specificity: Untangling the Relationship between Leishmania (Viannia) Species and Its Endosymbiont Leishmania RNA Virus 1
Source: Microorganisms. 2023 Sep 12;11(9):2295. doi: 10.3390/microorganisms11092295 (PMC10535429; doi:10.3390/microorganisms11092295)
Supplement: Supplementary file 1 [file microorganisms-11-02295-s001.zip › microorganisms-2573940-supplementary.pdf]

Table S1. Information on LRV1 sequences from previous studies used for phylogenetic analyses.

CLIOC *Leishmania* Strain ID (IOCL)	GenBank Accession Number	International Code	Parasite Species	Geographic Origin	Reference
3562	MG202146	MHOM/BR/2014/308	*L. braziliensis*	Porto Velho/Rondônia/Brazil	Cantanhêde et al., 2018 [21]
NA	MG202144	MHOM/BR/2015/386	*L. braziliensis*	Porto Velho/Rondônia/Brazil	Cantanhêde et al., 2018 [21]
3545	MG202143	MHOM/BR/2014/275	*L. braziliensis*	Porto Velho/Rondônia/Brazil	Cantanhêde et al., 2018 [21]
3569	MG202145	MHOM/BR/2014/299	*L. braziliensis*	Porto Velho/Rondônia/Brazil	Cantanhêde et al., 2018 [21]
3567	MG202139	MHOM/BR/2014/291	*L. braziliensis*	Porto Velho/Rondônia/Brazil	Cantanhêde et al., 2018 [21]
3538	MG202140	MHOM/BR/2014/271	*L. guyanensis*	Candeias do Jamari/Rondônia/Brazil	Cantanhêde et al., 2018 [21]
3354	MG202141	MHOM/BR/2011/S77-ABF	*L. guyanensis*	Santarem/Pará/Brazil	Cantanhêde et al., 2018 [21]
1545	MG202142	MCEB/BR/1984/M8408	*L. shawi*	Paraupebas/Pará/Brazil	Cantanhêde et al., 2018 [21]
3460	MG202147	MHOM/BR/2013/04LTAVMR	*L. guyanensis*	Manaus/Amazonas/Brazil	Cantanhêde et al., 2018 [21]
3461	MG202148	MHOM/BR/2013/05LTAMVL	*L. guyanensis*	Manaus/Amazonas/Brazil	Cantanhêde et al., 2018 [21]
3486	MG202149	MHOM/BR/2013/27JNS	*L. guyanensis*	Manaus/Amazonas/Brazil	Cantanhêde et al., 2018 [21]
3542	MG202151	MHOM/BR/2014/233CFS	*L. guyanensis*	Manaus/Amazonas/Brazil	Cantanhêde et al., 2018 [21]
3503	MG202150	MHOM/BR/2013/33LGS	*L. guyanensis*	Manaus/Amazonas/Brazil	Cantanhêde et al., 2018 [21]
1398	JX313127	MHOM/BR/1989/IM3597	*L. guyanensis*	Manaus/Amazonas/Brazil	Cantanhêde et al., 2018 [21]
NA	KY750607	MHOM/FG/2011/2001	*L. guyanensis*	Manaus/Amazonas/Brazil	Tirera et al., 2017 [22]
565	KX808487	MHOM/BR/1975/M4147	*L. guyanensis*	Monte Dourado/Pará/Brazil	Brettmann et al., 2016 [34]

565	U01899	MHOM/BR/1975/M4147	*L. guyanensis*	Monte Dourado/Pará/Brazil	Tirera et al., 2017 [22]
NA	JX313126	IWHI/BR/1978/M5313	*L. guyanensis*	Monte Dourado/Pará/Brazil	Tirera et al., 2017 [22]
NA	KY750608	MHOM/FG/2013/LF94	*L. guyanensis*	French Guiana	Tirera et al., 2017 [22]
NA	KY750609	MHOM/FG/2013XJ93	*L. guyanensis*	French Guiana	Tirera et al., 2017 [22]
NA	KY750610	MHOM/FG/2013YA70	*L. braziliensis*	French Guiana	Tirera et al., 2017 [22]
NA	KY750611	MHOM/FG/2013/2014	*L. guyanensis*	French Guiana	Tirera et al., 2017 [22]
NA	KY750612	MHOM/FG/2012/2008	*L. guyanensis*	French Guiana	Tirera et al., 2017 [22]
NA	KY750613	MHOM/FG/2012/2015	*L. guyanensis*	French Guiana	Tirera et al., 2017 [22]
NA	KY750614	MHOM/FG/2012/2028	*L. guyanensis*	French Guiana	Tirera et al., 2017 [22]
NA	KY750615	MHOM/FG/2012/2028	*L. guyanensis*	French Guiana	Tirera et al., 2017 [22]
NA	KY750616	MHOM/FG/2012/2028	*L. guyanensis*	French Guiana	Tirera et al., 2017 [22]
NA	KY750617	MHOM/FG/2013/LF98	*L. guyanensis*	French Guiana	Tirera et al., 2017 [22]
NA	KY750618	MHOM/FG/2012/LL28	*L. guyanensis*	French Guiana	Tirera et al., 2017 [22]
NA	KY750619	MHOM/FG/2012/MC71	*L. guyanensis*	French Guiana	Tirera et al., 2017 [22]
NA	KY750620	MHOM/FG/2012/MJ25	*L. guyanensis*	French Guiana	Tirera et al., 2017 [22]
NA	KY750621	MHOM/FG/2014/PD46	*L. guyanensis*	French Guiana	Tirera et al., 2017 [22]
NA	KY750622	MHOM/FG/2012/VL19	*L. guyanensis*	French Guiana	Tirera et al., 2017 [22]
NA	KY750623	MHOM/FG/2013/VW21	*L. guyanensis*	French Guiana	Tirera et al., 2017 [22]

NA	KY750624	MHOM/FG/2012/WF69	*L. guyanensis*	French Guiana	Tirera et al., 2017 [22]
NA	KY750625	MHOM/FG/2012/WF69	*L. guyanensis*	French Guiana	Tirera et al., 2017 [22]
NA	KY750626	MHOM/FG2013/XJ93	*L. guyanensis*	French Guiana	Tirera et al., 2017 [22]
NA	KY750627	MHOM/FG/2013/XK73	*L. guyanensis*	French Guiana	Tirera et al., 2017 [22]
NA	KY750628	MHOM/FG/2013/YE48	*L. guyanensis*	French Guiana	Tirera et al., 2017 [22]
NA	KY750629	MHOM/FG/2014/YR07	*L. guyanensis*	French Guiana	Tirera et al., 2017 [22]
NA	KY750630	MHOM/FG/2012/YZ58	*L. guyanensis*	French Guiana	Tirera et al., 2017 [22]
NA	KX808483	MHOM/BO/1990/AN	*L. braziliensis*	Bolivia	Brettmann et al., 2016 [34]
NA	KX808486	MHOM/BO/IMT252	*L. braziliensis*	Bolivia	Brettmann et al., 2016 [34]
NA	KX808485	MHOM/BO/1990/CS	*L. braziliensis*	Bolivia	Brettmann et al., 2016 [34]
NA	KX808484	MHOM/BO/1990/CS	*L. braziliensis*	Bolivia	Brettmann et al., 2016 [34]
NA	KC862308	MHOM/BO/2011/2169	*L. braziliensis*	Bolivia	Parmentier et al., 2016 [35]
NA	M92355	MHOM/SR/1980/CUMC1	*L. guyanensis*	Suriname	Parmentier et al., 2016 [35]

NA: Non applicated

Table S2. Estimates of average evolutionary divergence over sequence pairs within groups. The number of base substitutions per site from averaging over all sequence pairs within each group are shown. Analyses were conducted using the Tamura 3-parameter model. Codon positions included were 1st+2nd+3rd+Noncoding. All ambiguous positions were removed for each sequence pair (pairwise deletion option). Evolutionary analyses were conducted in MEGA X. The presence of n/c in the results denotes cases in which it was not possible to estimate evolutionary distances.

L. shawi	0,008
L. naiffi	0,052
***L. guyanensis* (A)**	0,075
***L. guyanensis* (B)**	0,056
***L. guyanensis* (C)**	n/c
***L. guyanensis* (D)**	0,071
***L. guyanensis* (E)**	0,033

L. guyanensis **(A, B, C)**	0,115
L. braziliensis	0,132

Table S3. Estimates of evolutionary divergence over sequence pairs between groups using the Tamura 3-parameter model. The number of base substitutions per site from averaging over all sequence pairs between groups are shown. This analysis involved 59 nucleotide sequences. Codon positions included were 1st+2nd+3rd+Noncoding. All positions containing gaps and missing data were eliminated (complete deletion option).

	L. shawi	*L. naiffi*	*L. guyanensis* **(B)**	*L. guyanensis* **(A)**	*L. guyanensis* **(C)**	*L. guyanensis* **(E)**	*L. guyanensis* **(D)**	*L. braziliensis*
L. shawi								
L. naiffi	0,17							
L. guyanensis **(B)**	0,13	0,18						
L. guyanensis **(A)**	0,15	0,17	0,11					
L. guyanensis **(C)**	0,12	0,15	0,10	0,11				
L. guyanensis **(E)**	0,17	0,18	0,18	0,18	0,16			
L. guyanensis **(D)**	0,18	0,18	0,19	0,18	0,18	0,18		
L. braziliensis	0,20	0,17	0,21	0,20	0,19	0,19	0,20	

Table S4. Estimates of evolutionary divergence over sequence pairs between groups using the Tamura 3-parameter model. The number of base substitutions per site from averaging over all sequence pairs between groups are shown. Analyses were conducted using the Tamura 3-parameter model. This analysis involved 59 nucleotide sequences. Codon positions included were 1st+2nd+3rd+Noncoding. All positions containing gaps and missing data were eliminated (complete deletion option). There were a total of 279 positions in the final dataset.

	L. shawi	*L. naiffi*	*L. guyanensis* **(A, B, C)**	*L. guyanensis* **(E)**	*L. guyanensis* **(D)**	*L. braziliensis*
L. shawi						

L. naiffi	0,17				
***L. guyanensis* (A, B, C)**	0,14	0,17			
***L. guyanensis* (E)**	0,17	0,18	0,18		
***L. guyanensis* (D)**	0,18	0,18	0,19	0,18	
L. braziliensis	0,20	0,17	0,20	0,19	0,20
